# SARS-CoV-2 Placentitis and Intraparenchymal Thrombohematomas Among COVID-19 Infections in Pregnancy

**DOI:** 10.1001/jamanetworkopen.2022.5345

**Published:** 2022-03-21

**Authors:** Anh Huynh, Jennifer K. Sehn, Ilona Telefus Goldfarb, Jaclyn Watkins, Vanda Torous, Amy Heerema-McKenney, Drucilla J. Roberts

**Affiliations:** 1 Department of Pathology, Massachusetts General Hospital, Boston; 2 Department of Pathology, St Louis University School of Medicine, St Louis, Missouri; 3 Division of Maternal Fetal Medicine, Department of Obstetrics & Gynecology, Massachusetts General Hospital, Boston; 4 Pathology and Laboratory Medicine Institute, Cleveland Clinic, Cleveland, Ohio

## Abstract

This cases series examines SARS-CoV-2 placentitis and intraparenchymal thrombohematomas among COVID-19 infections during pregnancy.

## Introduction

Systematic meta-analysis did not find increased stillbirth risk during the early months of the SARS-CoV-2 pandemic,^1^ but increased rates have been reported during the Delta wave.^2,3^ Pathology literature has documented diagnostic histopathologic features of SARS-CoV-2 placental infection: syncytiotrophoblast necrosis, increased perivillous fibrin, and intervillositis, with trophoblast infection confirmed by SARS-CoV-2 RNA in situ hybridization (RNA-ISH) or immunohistochemistry.^4^ SARS-CoV-2 placentitis causes severe placental damage, resulting in perinatal morbidity and mortality.^3,5^ Some variants of concern appear more perinatally virulent.^5,6^ We study placental pathology and perinatal outcomes in a series of SARS-CoV-2 placentitis cases before and during the Delta wave.

## Methods

With Massachusetts General Hospital (MGH) institutional review board approval (consent-exempt because this study used excess human material), a series of placentas with a histopathologic diagnosis of SARS-CoV-2 placentitis reviewed at MGH Pathology Department from January 1, 2020, through November 4, 2021, was retrospectively reviewed. Confirmatory histopathology and placental SARS-CoV-2 RNA-ISH were present in all placentitis cases using previously published methods.^4^ Clinical data were manually abstracted from the electronic medical record or obtained from the referring physician. Histopathologic and clinical features of cases from 2020 (pre-Delta) and 2021 (Delta wave) were compared, as were cases with intraparenchymal thrombohematomas vs without. Data were stored in Microsoft Excel spreadsheets. This study followed applicable sections of the Strengthening the Reporting of Observational Studies in Epidemiology (STROBE) reporting guideline. Strain data was obtained as previously described.^5^

## Results

At MGH, all SARS-CoV-2 exposed placentas were fully pathologically examined. Of 40 placentas with neonate sex available, 24 were female. Forty-seven cases of SARS-CoV-2 placentitis were identified by histopathologic criteria and confirmed by SARS-CoV-2 RNA-ISH (Figure), 39 from 2021 (6 previously published^3,5^) and 8 from 2020 (Table). These included 15 in-house MGH cases and 32 referred for consultation. In all cases, the placentitis was diffuse (>50% involvement). Twenty-nine of the 47 cases (62%), all from 2021, additionally had multiple intraparenchymal thrombohematomas (Figure). Of these, 21 (72%) were stillbirths. Seventeen of the 18 placentas without thrombohematomas were livebirths (94%). Viral strain was identified in 8 cases: 3 Delta (2 stillbirths with thrombohematomas, 1 livebirth without thrombohematomas^5^), 2 Alpha, and 3 other strains, all livebirths without thrombohematomas. In 2 cases the ultrasound documenting fetal demise demonstrated multiple large maternal lakes with echogenic rims. When known, SARS-CoV-2 infection was most commonly diagnosed within 14 days before delivery (29 of 33 cases; 88%). In 2 cases, the mother was untested and had no documented infection or symptoms. All patients but 1 were unvaccinated. The vaccinated patient (from 2021) had a PCR-confirmed infection 7 weeks prior to delivery and was subsequently vaccinated once. It is unclear if she cleared her virus or completed her vaccination series. Her term livebirth was without thrombohematomas.

**Figure.**
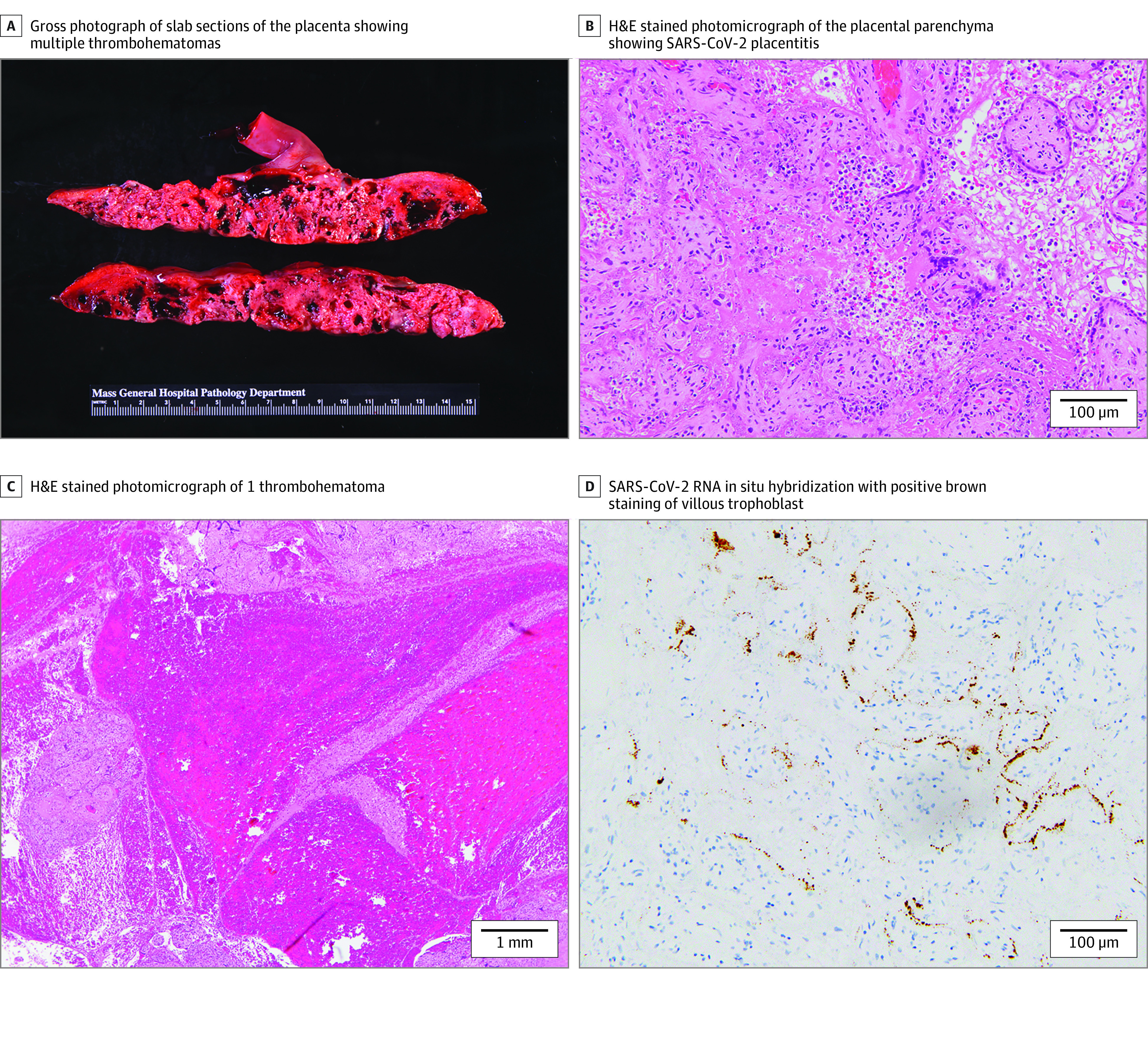
SARS-CoV-2 Placentitis With Thrombohematomas From a 37.1 Week Gestational Age Intrauterine Demise A, Gross photograph of slab sections of the placenta showing multiple thrombohematomas (dark red). B, Hematoxylin and eosin (H&E) stained photomicrograph of the placental parenchyma showing the triad of SARS-CoV-2 placentitis: histiocytic intervillositis, increased perivillous fibrin, and villous trophoblast necrosis at 200× original magnification. C, H&E stained photomicrograph of 1 thrombohematoma at 20× original magnification. D, SARS-CoV-2 RNA in situ hybridization with positive brown staining of villous trophoblast at 200× original.

**Table.** Clinical Characteristics of 47 Patients With SARS-CoV-2 Placentitis From January 1, 2020, to November 4, 2021

Characteristics	No. (%)						
	Thrombohematomas, 2021						Thrombohematomas absent, 2020 (n = 8)^a^
	Present (n = 29)			Absent (n = 10)			
	Stillbirth	Morbidity other than stillbirth^b^	No morbidity or mortality^c^	Stillbirth	Morbidity other than stillbirth^b^	No morbidity or mortality^c^	No morbidity or mortality^c^
All	21 (72)	2 (7)	6 (21)	1 (10)	4 (40)	5 (50)	8 (100)
Maternal age							
Mean (range), y	29 (18-41)	29 (26-32)	28 (18-31)	34 (34)	30 (23-33)	27 (20-33)	28 (18-40)
Not available, No.	1	0	0	0	0	0	0
Gestational age at delivery							
Mean (range), wk + d	29 + 0 (17 + 0 to 37 + 1)	30 + 3 (28 + 6 to 32 + 0)	34 + 2 (32 + 0 to 38 + 5)	34 + 6 (34 + 6)	28 + 1 (23 + 2 to 29 + 4)	35 + 0 (29 + 1 to 39 + 2)	36 + 0 (32 + 0 to 39 + 5)
Not available, No.	1	0	0	0	0	0	0
Time from COVID-19 positive test or symptoms to delivery, d							
0-14	14 (48)	2 (7)	3 (10)	0	3 (30)	2 (30)	5 (75)
>14	1 (3)	0	1 (3)	0	0	2 (20)	0
Not available	6 (21)	0	2 (7)	1 (10)	1 (10)	1 (10)	2 (25)
Maternal COVID-19 symptoms peripartum, No.^d^							
Asymptomatic	7	0	1	0	3	2	1
Mild	8	0	1	0	1	2	4
Severe	0	2	1	0	0	0	0
Not available	6	0	3	1	0	1	2
Indications for delivery^e^							
IUFD	21 (72)	0	0	1 (10)	0	0	0
NRFHT or BPP < 4	0	1 (3)	5 (17)	0	2 (20)	2 (20)	2 (25)
Preeclampsia or (atypical) HELLP	0	1 (3)	1 (3)	0	0	2 (20)	1 (13)
Other, No. (%) [description]	0	1 (3) [critical maternal respiratory status]	0	0	2 (20) [abruption, preterm labor]	1 (10) [invasive PAS]	1 (13) [preterm labor]
Unknown, No.	0	0	0	0	0	1	3
Sex of the neonate							
Female	11 (38)	2 (7)	1 (3)	0	3 (30)	3 (30)	4 (50)
Male	7 (24)	0	2 (7)	1 (10)	1 (10)	2 (20)	3 (38)
Not available	3 (10)	0	3 (10)	0	0	0	1 (13)

Abbreviations: HELLP, Hemolysis, elevated liver enzymes, low platelets; IUFD, intrauterine fetal demise; NRFHT, nonreassuring fetal heart tracing; PAS, placental accreta spectrum disorder.

^a^ Includes 1 placenta from dichorionic-diamniotic twins.

^b^ This category includes cases with severe complications such as respiratory distress requiring intubation, early neonatal death, or intraventricular hemorrhage.

^c^ This category may include cases with complications of prematurity not previously specified as severe.

^d^ Mild symptoms include cough, fever, myalgias, chills, loss of taste and smell, nausea and vomiting, and diarrhea. Severe symptoms include pneumonia and respiratory distress requiring intensive care unit admission and/or intubation.

^e^ There may be multiple indications for each delivery.

## Discussion

We describe a severe form of SARS-CoV-2 placentitis with thrombohematomas occurring primarily in stillbirths from pregnancies complicated by SARS-CoV-2 infection during the 2021 pandemic wave. This pathology is distinctive and grossly identifiable, representing a change in the spectrum of SARS-CoV-2 pregnancy complications.^1,2^ The thrombohematomas are likely a result of severe viral placental damage. Our findings suggest a pathogenetic mechanism for the reported increased risk of stillbirth associated with SARS-CoV-2 infection in 2021.^2,3^ As maternal lakes were noted by ultrasound in 2 cases, further research is needed to understand if imaging may identify at-risk pregnancies.

Our study has several limitations, including (1) ascertainment bias due to inclusion of consultation cases and lack of universal SARS-CoV-2 screening at referring institutions, which limits assessment of the incidence of SARS-CoV-2 placentitis and stillbirth; (2) inability to obtain outcome data in several consultation cases; and (3) placental sonography was not reported after SARS-CoV-2 infection in most cases, and strain identification was limited.

Updated epidemiological studies evaluating pregnancy outcomes are required for corroboration of our findings. Follow-up studies of patients affected by SARS-CoV-2 placentitis are required for evaluation of long-term clinical outcomes.

## References

[1] Huntley BJF, Mulder IA, Di Mascio D, . Adverse pregnancy outcomes among individuals with and without severe acute respiratory syndrome coronavirus 2 (SARS-CoV-2): a systematic review and meta-analysis. Obstet Gynecol. 2021;137(4):585-596. doi:10.1097/AOG.0000000000004320 33706357 PMC7984633

[2] DeSisto CL, Wallace B, Simeone RM, . Risk for stillbirth among women with and without COVID-19 at delivery hospitalization - United States, March 2020-September 2021. MMWR Morb Mortal Wkly Rep. 2021;70(47):1640-1645. doi:10.15585/mmwr.mm7047e1 34818318 PMC8612508

[3] Schwartz DA, Avvad-Portari E, Babál P, . Placental tissue destruction and insufficiency from COVID-19 causes stillbirth and neonatal death from hypoxic-ischemic injury: a study of 68 cases with SARS-CoV-2 placentitis from 12 countries. Arch Pathol Lab Med. 2022. doi:10.5858/arpa.2022-0029-SA 35142798

[4] Watkins JC, Torous VF, Roberts DJ. Defining severe acute respiratory syndrome coronavirus 2 (SARS-CoV-2) placentitis. Arch Pathol Lab Med. 2021;145(11):1341-1349. doi:10.5858/arpa.2021-0246-SA 34338723

[5] Shook LL, Brigida S, Regan J, . SARS-CoV-2 placentitis associated with B.1.617.2 (Delta) variant and fetal distress or demise. J Infect Dis. Published online January 13, 2022. doi:10.1093/infdis/jiac008 35024844 PMC8807229

[6] Royal College of Physicians of Ireland FoPatIoOaG. Covid placentitis: statement from the RCPI Faculty of Pathology and the Institute of Obstetricians and Gynaecologists. Accessed February 23, 2022. https://www.rcpi.ie/news/releases/covid-placentitis-statement-from-the-rcpi-faculty-of-pathology-and-the-institute-of-obstetricians-and-gynaecologists/

